# A Place without Doctors

**Published:** 1847-09

**Authors:** 


*A place without Doctors.*—The following annunciation, which we copy from the Western Lancet, is certainly, as the Editor observes, extraordinary. Wonders never cease! We may now hope that the philosopher's stone will be discovered! We shall expect shortly to receive instructions from some of our subscribers to change the direction of the Journal to *Geneva, Coffee County, Alabama*, the place without doctors. The Post-master from said place thus writes:—"Strange to say, among a population voting about eight hundred, some pretty large families, and this village containg about forty families, we cannot count *one doctor*. Our village is on a stream navigable for steamboats, at the heart of navigation, and in a cotton region." We trust in mercy that the place will be speedily supplied with doctors, lest the discovery be made that life and health are secure without them!

